# Management of Children in the Dental Office

**Published:** 1903-01

**Authors:** Horace E. Eaton

**Affiliations:** Toronto, Canada


### MANAGEMENT OF CHILDREN IN THE DENTAL OFFICE.[1]

BY DR. HORACE E. EATON, TORONTO, CANADA.

THE importance of caring for the teeth of the child is so well known and recognized to-day that I need not trespass upon your time by pointing out the many benefits arising from it. Much has been written regarding the treatment and filling of children's teeth, but in this paper it is my purpose to deal only with the matter of managing the children themselves in such a way as to secure the most favorable conditions for satisfactory work. I do not propose to formulate any fanciful theory which would fail to work out in practice, for I do not wish to infringe upon the rights of those elderly maidens and childless wives who so kindly acquaint the public through the ladies' journals with their unbounded knowledge of child management in all its details. I shall endeavor to give you a simple record of the results obtained in my own practice while following out a few fundamental principles.

Am I not correct when I say that the average practitioner prefers an adult to a child for his patient? With the former he can, as a rule, accomplish more in a given time, and more satisfactorily. But is it less important that the child should have the very best treatment it is possible to give? Emphatically, no! It becomes our duty, then, to study ways and means, for I consider the successful management of the child as less important only than the care of its teeth. A dentist becomes a public benefactor when he can so successfully manage the child as to prevent or lessen its fear of dental operations, since even a single instance of fright or terror may have a lasting and harmful effect upon early mental development. The result from a professional standpoint is that operations can be more successfully performed and the child's teeth more regularly attended to.

Let me state, at the outset, that in order to achieve the greatest success in the management of the child, one should love children. If you do not possess this qualification, then cultivate it. It is your passport to the confidence of the child, which in turn is the first step towards its successful management. And the average child

---

[1] Read before the Massachusetts Dental Society, June 4, 1902.

is very quick to discern whether you have your passport. In attempting to form the acquaintance with and gain the confidence of the child the mistake is often made by us of proceeding from a grown-up stand-point instead of from that of the child. The child at once pictures the dentist in its mind as some awful, dignified creature that does not belong to its world. Thus a barrier is formed preventing further acquaintance. Let me say, in this connection, that the average child draws a marked line of distinction between a dentist and an ordinary family man; and in many cases with a prejudice against the dentist which has to be taken into consideration. To illustrate. A bright little girl about five years of age, when dining with us, fastened her eyes intently upon me, and asked, "Are you a father?" "Yes," I answered. "Are you a doctor?" I replied that I was. "Well," said she, "I don't see how you can be a father and a doctor too." To the child's mind the father was a human being, but the doctor—well, he was just a doctor, and perhaps an inhuman being.

When the child comes to the dentist for the first time, in the majority of cases you may be sure it has some conception of a dentist and his business, and that conception is formed usually from hearing injudicious remarks made by older members of the family. Therefore, we have first to overcome whatever prejudice there may be in its mind. In order to accomplish this, do not think your time too valuable to spend a few moments chatting with the little one. In this way you will probably discover some subject of special interest that you may afterwards use to good advantage in the management of the child. As a result of such conversations little girls have frequently brought their dolls for my inspection, and boys their boats and other toys, because I had exhibited an interest in these things during our conversations.

In placing a child—especially a nervous one—in the chair for the first time, make it a point to draw the attention, as far as possible, from anything that suggests work upon the teeth, until that timid, open-eyed watchfulness—expecting every moment to see the Jack pop out of the box—passes off. If it can possibly be arranged, do not have the mother accompany the child, for she is often the harder of the two to manage. In nine cases out of ten she will begin by saying something like this: "Now, it won't hurt you—you needn't be frightened, mother is right here." Then, as a side remark, she will tell how very nervous the child is,—"just like her mother,"—and in the same breath will relate some harrowing tale of her own experience in the dental chair. All this you have to tolerate or cut short as your wisdom directs you.

As a rule, it is unwise to inform the child at the beginning as to whether or not the operation will be a painful one, unless it may be in a case of extraction where you know it will be painful. In such a case it is better to tell the child frankly that it will probably hurt a little, but assuring it that you will be as gentle as possible. But never say it will not hurt when you know that it must, for in the majority of cases the child has learned from the inconsistencies of its parents not to take seriously what you say about the matter. Never, under any circumstances, deceive a child.. I have heard more than once of a dentist concealing the forceps up his sleeve, and asking the little patient to just let him look at the tooth. With the assurance that he would not take it out, he would then slip the forceps on and make the extraction. A man can do this but once before losing the confidence of the child and making it fear everything in connection with dentistry. It is supposed by some, I believe, to be clever. It is not. It is wicked, and should be denounced in the strongest terms.

Most children are imaginative to a wonderful degree, and this can be turned to excellent account. I sometimes liken the chair to an elevator, and ask them if they would like to go up to the toy department, or as I elevate the chair I make allusion to the story of "Jack and the Bean Stalk," which most children have had read to them, and they very much enjoy the "Hitch my toe, and up I go." It is surprising how quickly through their fanciful imaginations they enter into the spirit of it all.

The first step gained,—viz., the fear of the place and the fear of the dentist overcome,—I proceed to examine and excavate the cavity. By this time I have become so far acquainted with my little patient as to have discovered the direction of some strong inclination or hobby, which, as I have already hinted, I follow up. For example, a little patient of mine, I soon discovered, was passionately fond of fairy stories,—in fact, she was so absorbed by them that she seemed to live in fairy-land all the time. Taking advantage of this and following out a suggestion of her own,—that a family of fairies had made a house in her tooth,—I said, taking up an excavator, "Now, I'll just take this little fairy axe and break into the house and drive them out. You listen to them jump from their beds when they hear the noise on the roof." And so I got the walls of the cavity broken down and the excavation well proceeded with, much to the amusement and enjoyment of my little patient. Then, wishing to use the bur, I remarked, "I'm going to take this fairy broom now, and sweep the house out clean, fairies and all." I proceeded to drill, and any pain connected with it I blamed upon the fairy family rushing around to escape the broom, and upon their being thrown out by it. After the house was rid of the fairies and the floor swept clean, I repaired the hole I had made in the roof and the tooth was filled.

Another case. A little chap, who is a soldier from the ground up, made the remark to me one day as he seated himself in the chair, "The enemy have intrenched themselves in my teeth; what had we better do about it?" I answered that I thought we had better make an attack upon their fortifications. His enjoyment and interest were intense when I advanced with picks, shovels, and machine drills, and finally compelled the enemy to surrender. The breeches in the fort were then repaired and our men placed on duty to guard against a return of the enemy. I put him in command of the fort, with orders to go carefully over the outside walls with a tooth-brush and dentifrice every night and morning to clear away any of the enemy that might be lurking around. So I succeeded not only in making the operation a pleasure to him, but in getting him to take an interest in keeping his teeth clean. He calls me the inspector of fortifications.

Still another case. I found it necessary to use the engine in the preparation of a cavity for a timid little girl. I scarcely knew how to manage it, for the slightest thing seemed to alarm her. Finally an idea came to me. I placed a bur in the handpiece of my engine and said, "Have I ever shown you this musical instrument?" She said I had not. "Well," I said, "I'll play a tune for you on your tooth, and see if you can discover what tune it is." I began, charging her over and over again to pay very strict attention to the music. When the cavity was prepared, I said, "There, I have played one verse for you, do you know what tune it was?" "Yes," she replied, quite pleased with herself, "it was 'Soldiers of the Queen'." Of course she had a Canadian ear. To a child on this side of the line the same music might have been interpreted as "Yankee Doodle."

I once suggested to a little boy that we should go for a ride on a tandem wheel, and offered him his choice of seats. He chose the front. "Then," I said, "you will have to do the steering," which, of course, pleased him very much. I placed his hands each upon an arm of the chair, which served as the handle-bars. Everything ready, the engine hand-piece in my hand, I gave the word to start. As I drilled at his tooth, I called his attention to the roughness of the pavement and the rumble of the wheel, and cautioned him to be very careful about passing people, so that we might have no accident. I also called off the streets as we passed them, and thus kept his mind well employed while I was getting the cavity prepared. When we arrived at our destination he was so delighted with the trip that he wanted to turn around at once and take it over again. I could name dozens of other cases where, by taking advantage of the child's power of imagination and love of stories, I have succeeded in changing its thought of terror to one of pleasure. In some cases the child would even pretend to its mother that there was something wrong with its teeth, as an excuse to be taken to the dentist.

Now, let us for a moment consider the foregoing illustrations. What has taken place? Simply this: A pleasing and interesting picture has been suggested to the child's mind, stimulating the imagination and occupying the whole of the attention. The brain, being thus occupied, cannot attend at the same time to other calls; hence the sensations produced by the operation are greatly diminished. Again, the pleasure produced by the suggested thought is so intimately associated with the work being done that the operation itself becomes transformed into a pleasant experience. Ruskin tells us that when young he associated the name "crocodile" with the creature so closely that the long series of letters took on something of the look of its lanky body. The same writer speaks of a Dr. Grant, into whose therapeutic hands he fell when a child. "The name," he adds, "is always associated in my mind with a brown powder—rhubarb, or the like—of a gritty or acrid nature." We can, most of us, perhaps, recall similar experiences, where colors or sounds, in themselves indifferent, took on, through analogy and association, a decidedly repulsive or decidedly pleasurable character. In dealing with children in the ways I have described, the object is gained of making the story or suggested thought the prominent feature of the visit in the child's mind. Filling the teeth becomes a mere incident. To further illustrate this point. A little chap came into my office one day with this remark: "Dr. Eaton, I've come back to hear the rest of that story."

I conclude, then, that a very large part of the disagreeableness in connection with a dental operation is due to the picture that is previously formed of it in the patient's mind. For instance, have you not all experienced the following? In beginning the preparation of a cavity your patient flinches and cringes until you assure him that the cavity is a small one not near the nerve, or tell him it is not a sensitive part of the tooth. Immediately he quiets down and allows you to proceed with the work. It seems strange that it should be necessary in certain cases to inform one's patient that this or that part is not sensitive, when he should be the first to find it out. But the explanation is this. So vividly has he imagined it is going to hurt that it is necessary to inform him to the contrary before he can realize that it does not. Ask a boy to run around the block to do an errand for you. He does not want to go,—he is tired; but five minutes later another boy comes along and asks him to join in a paper chase or game of tag. He at once accepts the invitation and probably runs almost constantly for the next two hours. How may this be accounted for? By the difference in the pictures formed in the boy's mind,—the one is pleasing to him, the other is not.

Thus far I have dealt only with one class of children, the imaginative sort. But there are those who have no imagination whatever, and to whom the suggestion I have offered would not in any sense apply. These, however, as well as other children, have human nature enough to enjoy a little of what is commonly called "taffy." For instance, if I say, as I begin work, "Why, how wonderfully still you keep! And how wide you can hold your mouth open! Now, some children would be talking and twisting about," it is amusing to see the effort that will be put forth to maintain this reputation.

Then, we meet with other children who study to oppose in every possible way. These are not always hopeless cases. Perhaps illustration will best describe the handling of such. I once attempted to insert a cement filling in the mouth of a little girl aged about five years. Applying the rubber dam was out of the question, and the trick was to keep the cavity dry long enough to get the cement in,—for as soon as she discovered that I was anxious to keep it dry, she was just as anxious to get it wet. After an unsuccessful attempt, I said to my assistant, "Now, *I* say she will get it wet this time; what do *you* say?" "I say the same." Determination to keep it dry could be seen in her face, and as I proceeded I remarked, "If she would only begin to talk and get that cavity wet, then we would be right." But no, her tongue remained motionless until the operation was completed, when I announced, to her great satisfaction, that we were wrong. Another little patient brought by her mother would not sit in the chair. Instead of coaxing her, I had her mother take the chair, and proceeded as if doing work for her. The child standing in front of her, defiantly looked at me and said, "I will not let you fix my teeth." I replied, "Well, I haven't time to attend to yours to-day, anyway; I am too busy,—couldn't do it if you wished me to." She immediately changed her attitude and wanted her teeth fixed. Of course, I reluctantly consented to wait upon her.

We have also the spoiled child to deal with,—one who has become the lord of the home, and thinks he is privileged to exercise the same right wherever he goes. This child needs special treatment. Great care must be exercised lest he become so well acquainted with you that you entirely lose control over him. Playing with him is often dangerous. Firmness, with kindness, must be used, but never use force to compel a child to undergo an operation, although you may often be asked by the mother to do so. Let me say, with double emphasis, *never, under any circumstances, lose your temper with the child.* This sometimes will require an abundance of grace.

I wish to make myself clearly understood with regard to the methods of management that I have suggested. They are not play, though they may appear so to the child, for I introduce nothing that will not in some way serve my purpose. I proceed on a definite plan, with definite principles of which I never lose sight. While the particular methods I have described may apply only to children of certain dispositions, I trust that they afford some illustration of underlying principles that are applicable in every case. I shall be satisfied if I have thrown out some suggestions that may stimulate interest and thought along the line of making it easier for children to receive proper dental treatment, and more pleasant for operators to give it. The importance of getting children started right can hardly be over-estimated. It is to their experience with dentistry what the kindergarten is to their education,—it carries them over a very trying period in a very enjoyable way.

As we are the fathers of the next generation of dentists, so these little ones are to be their future patients. Let us face the responsibility that is surely ours, and hand over to them patients whose minds are rid of any false dread of the dental chair. By so doing we shall remove in a large measure that popular antipathy to everything connected with dentistry that to-day hampers the profession in its efforts to benefit those whom it endeavors to serve.

